# Sexual dimorphism in zebrafish liver proteins and implications for hepatic regeneration and diseases

**DOI:** 10.1038/s41598-025-18599-2

**Published:** 2025-09-29

**Authors:** Hamid Niksirat, Kifayatullah Mengal, Golara Kor, Christoph Steinbach, Fredrik Levander

**Affiliations:** 1 Faculty of Fisheries and Protection of Waters, University of South Bohemia in České Budějovice, CENAKVA, Zátiší 728/II, 38925 Vodňany, Czech Republic; 2 School of Ecology, Sun Yat-sen University, Shenzhen, China; 3 School of Fisheries, Aquaculture, and Aquatic Sciences, Auburn University, Alabama, USA; 4 Department of Immunotechnology, Science for Life Laboratory, Lund University, Lund, Sweden; 5 Science for Life Laboratory, National Bioinformatics Infrastructure Sweden (NBIS), Lund University, Lund, Sweden

**Keywords:** Sexual dimorphism, Liver proteomics, Zebrafish model, Metabolism, Drug metabolism, Liver disease, Proteomics, Animal physiology, Liver, Proteome informatics

## Abstract

The liver is a central metabolic hub, performing vital functions such as bile production, protein, carbohydrate, lipid and drug metabolism, detoxification of xenobiotics, and the synthesis of essential biomolecules for reproduction, and also shows regenerative capability. Several of these functions can be affected by sexual dimorphisms with important consequences. In this study we used high-throughput proteomics to identify and quantify proteins involved in sexual dimorphism of the zebrafish liver, as a model for preclinical human research. Additionally, we conducted an extensive literature review to explore potential effects of sex-biased protein abundances on liver regeneration capacity and hepatic diseases. The results showed wide-spread sex-specific differences in proteins involved in carbohydrate, protein, and lipid metabolism. Female livers exhibited higher levels of proteins involved in protein synthesis, while male liver protein abundances were higher in energy-producing biochemical pathways, such as the TCA, β-oxidation, and glycolysis. Furthermore, significant sex differences were observed in proteins related to drug metabolism, which should be considered in toxicological and pharmacological research. Some potential links between sex-biased quantities of some key hepatic proteins and the susceptibility of males to liver diseases, as well as the higher hepatic regenerative capacity in females, were suggested. These findings offer a foundation for future targeted research to facilitate the development of sex-specific therapeutic approaches for liver disorders and regenerative medicine. Data are available via ProteomeXchange with identifier PXD061886.

**Supplementary Information:**

The online version contains supplementary material available at 10.1038/s41598-025-18599-2.

## Introduction

Sexual dimorphism programs structural, functional, and metabolic differences in non-sexual organs to support the reproductive needs of the male and female sexual organs, which produce small, motile spermatozoa and large, nutrient-rich oocytes, respectively. On the other hand, such differences can influence the capacity of each sex for lipid and nutrient metabolism, immunity, and drug metabolism^1–3^.

The liver, as a central metabolic hub, is responsible for a wide range of vital functions, including bile production for digestion, metabolism of proteins, carbohydrates, lipids and drugs, detoxification of xenobiotics, and synthesis of numerous proteins and lipids essential for reproduction^4^. Sexual dimorphism in the cellular and molecular characteristics of the liver can lead to an individual’s predisposition or protection against certain hepatic diseases in a sex-biased manner. For example, the prevalence of nonalcoholic fatty liver disease (NAFLD) and hepatocellular carcinoma (HCC) is higher in males^5,6^. In addition, females demonstrate a superior capacity for liver regeneration compared to males^7^.

Relatively easy maintenance and a high level of genetic similarity with humans made zebrafish a suitable model organism for human preclinical studies^8^. Their livers share conserved metabolic pathways with those of humans and can be used as a model for investigating human liver development, diseases, and regeneration^9^.

Zebrafish have been used to explore sexual dimorphism in the cardiovascular system^10^, ocular system^11^, and gastrointestinal tract^12^. Despite some earlier studies on sexual dimorphism in the liver of fish^13,14^ to our knowledge, no study has yet quantified sex-based differences in liver protein abundances in zebrafish. With proteins as functional molecules in the cell that orchestrate sexual dimorphism in the organ, such analyses could provide resolved information about underlaying mechanisms.

Therefore, the primary goal of the present study was to perform a comprehensive proteomic analysis to identify and quantify sexually dimorphic proteins and associated biochemical pathways in the zebrafish liver. We also performed an extensive literature search to explore the potential relevance of these sex-biased proteins for the differences observed between men and women in regeneration capacity and the prevalence of hepatic diseases.

## Methods

Livers were obtained from six male and six female two-year-old wild-type AB line adult zebrafish, originating from the University of Veterinary and Pharmaceutical Sciences Brno. Zebrafish were selected from fertile animals with functional spermatozoa or mature eggs, confirmed by prior successful fertility assessments. Animals were anesthetized in ice water and sacrificed by cutting their spinal cord using sharp scissors. Livers were removed and immediately plunged in liquid nitrogen (– 196 °C) and transferred to a – 80 °C freezer until processing for proteomics. All animal handling procedures were conducted with the approval of the Institutional Animal Care and Use Committee of the University of South Bohemia in České Budějovice. All methods were carried out in accordance with relevant guidelines and regulation. The present study is reported in accordance with ARRIVE guidelines (https://arriveguidelines.org).

### Protein extraction and digestion

Protein extraction and digestion were performed according to previously described protocols^15–17^. Livers were homogenized in 1% SDS, and protein concentrations were determined using the Micro-Lowry method. Protein samples underwent reduction and alkylation, before purification and digestion using hydrophilic interaction liquid chromatography beads and automated on-bead trypsin digestion in a KingFisher Flex system. Peptides were purified by C18 desalting and stored at − 20 °C until further analysis. A detailed step-by-step protocol is provided in the Supporting Information Materials (Supplementary Methods).

### Mass spectrometry

Protein digests were reconstituted in 0.1% formic acid loaded on Evotips (Evosep) according to the manufacturer’s instructions and separated using an Evosep One liquid chromatography (LC) system (Evosep) with a 15 cm analytical column. The analytical column consisted of a fused silica capillary (75 μm × 16 cm Pico Tip Emitter, New Objective, USA) packed in-house with ReproSil-Pur 1.9 μm C18 resin (Dr. Maisch GmbH, Germany), cut to a length of 15 cm. Peptide separation was performed using the Whisper 20 SPD method.

The LC system was connected with a Q Exactive HF-X mass spectrometer (Thermo Fisher Scientific, Germany), operating in positive ion mode with top-20 data-dependent acquisition. Full MS scans were collected in profile mode between 375 and 1500 m/z at a target resolution of 120,000 and an automatic gain control (AGC) target of 3 × 10⁶ ions with a maximum injection time of 50 ms. Precursor ions with charge states 2 –6 were selected for high-energy dissociation (HCD) fragmentation using a 1.2 m/z isolation window with a normalized collision energy of 27. The resulting MS/MS spectra were recorded in centroid mode at a resolution of 15,000 FWHM, with an AGC target of 100,000 ions and a maximum injection time of 20 ms.

### Data processing and analysis

The raw MS data files were processed using MaxQuant version 2.4.7.0^18^, with the UniProt Zebrafish protein database as of October 6, 2023. Match-between-runs was enabled, and the default settings were applied for modifications and missed cleavages. A 1% False Discovery Rate (FDR) filter was applied at the peptide level. Peptide data were normalized using Cyclic Loess in NormalyzerDE^19^followed by protein grouping using the RRollup method^20^with at least two peptides per protein group, using the implementation at https://github.com/ComputationalProteomics/ProteinRollup.

Protein-level abundance data were analyzed for differential abundance using an empirical Bayes t-test (Limma)^21^ in NormalyzerDE, followed by Benjamini − Hochberg adjustment of p-values (these adjusted p-values are referred to as q-values). Volcano and Principal Component Analysis (PCA) plots were generated using OmicLoupe^22^. Gene ontology analysis for the liver sex-biased proteins was performed via the ShinyGO (v0.8) webserver^23^. Gene Set Enrichment Analysis (GSEA)^24^ was conducted via the clusterProfiler R package^25^. The analysis incorporated the CP: KEGG gene set collection, ranking proteins according to their log2 fold change. For proteins with all missing values in one group, the log2 fold change was estimated as the difference between the mean of the log2 abundance values of the other group and the lowest abundance value found for any protein. The mass spectrometry proteomics data have been deposited to the ProteomeXchange Consortium via the PRIDE^26^ partner repository with the dataset identifier PXD061886. KEGG pathway^27–29^ visualization of protein abundance differences was performed using the KEGG Mapper^30,31^ Color tool after mapping protein accessions to Entrez IDs using org.Dr.eg.db (10.18129/B9.bioc.org.Dr.eg.db).

## Results and discussion

**Fig. 1:**
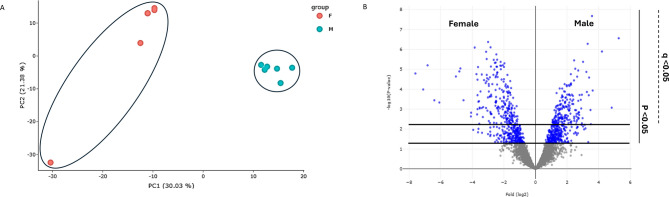
(**a**) Distribution of female (left) and male (right) zebrafish liver samples in a PCA plot showing separation of the sexes in the first principal component, (**b**) Volcano plot illustrating differentially abundant proteins in the livers of male and female zebrafish. The protein abundance ratio of male/female (log2 scale) in label-free protein quantitation was plotted against the − log10(p-value) of the probability calculated by the LIMMA t-test, with proteins passing *p* < 0.05 in blue. The significance threshold for FDR < 0.05 (q < 0.05) is also indicated.

A total of 3695 protein groups were identified in the zebrafish liver. Analysis of the data using PCA of quantified protein groups showed a clear separation between samples of the different sexes in the first principal component (Fig. 1a), and the protein abundance differences between proteins in the livers of female and male zebrafish appeared balanced in a volcano plot (Fig. 1b). The abundances of 404 protein groups exhibited statistically significant differences (FDR < 0.05) with 918 proteins passing unadjusted p-value < 0.05. Among these 404 proteins, 217 and 187 were more abundant in females and males, respectively. In addition, 114 and 9 unique proteins were exclusively detected in females and males, respectively (Supplementary Table 1).

**Fig. 2:**
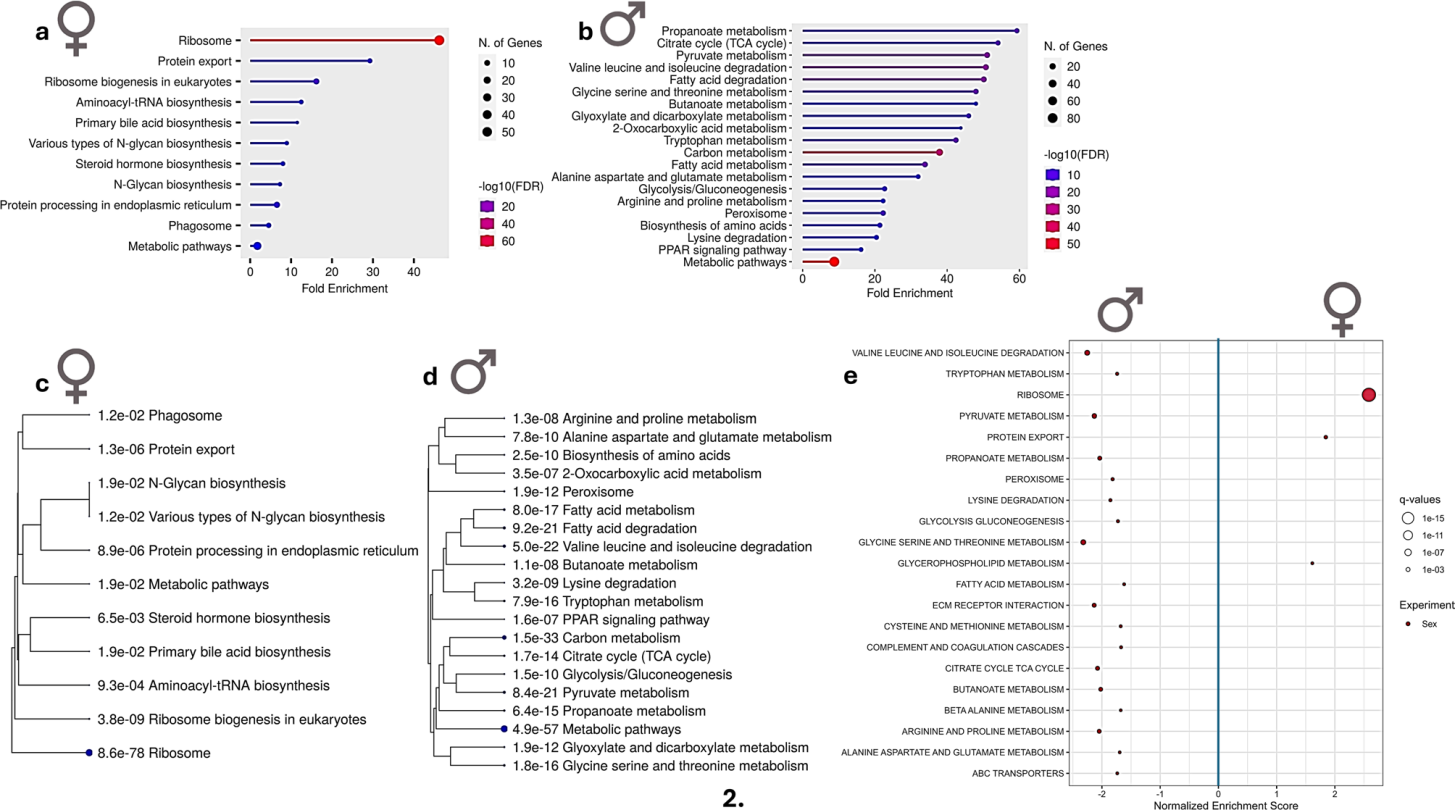
(**a**, **b**) Dot plots illustrating ShinyGO Gene Ontology (GO) enrichment analysis for KEGG sex-biased proteins in the liver of female (**a**) and male (**b**) zebrafish. Larger dots show pathways involving a greater number of proteins. (**c**, **d**) Hierarchical clustering trees provide an overview of the relationships among significant KEGG pathways for females (**c**) and males (**d**), grouping pathways that share numerous genes. The charts were generated using ShinyGO (v0.80). (**e**) Gene Set Enrichment Analysis (GSEA) using the KEGG gene set collection (FDR < 0.1). Positive and negative normalized enrichment scores denote enrichment in females and males, respectively.

Based on the protein differential abundance analyses, we categorized the proteins according to the enrichment of differences in functional pathways using multiple methods to find functional patterns (Fig. 2). These analyses highlighted several pathways which led us to explore the potential implications of sex-biased differences in liver protein abundance by further searching their known roles in hepatic regeneration and disease susceptibility. By integrating our findings with existing literature, we investigated how the differential abundances of proteins between male and female zebrafish may contribute to sex-specific variations in liver regeneration capacity and the predisposition to hepatic disorders in humans. Such approach can provide insights into potential biological factors underlying sex-related differences in liver pathology.

### Protein synthesis machinery and implications in sex-biased liver regeneration

The functional enrichment analyses showed a strikingly higher abundance of proteins involved in proteins synthesis, such as ribosomal function and protein transport in female zebrafish livers compared to males (Fig. 2a, c, e). When ribosomal proteins were mapped to a KEGG pathway map, almost all detected proteins were found at higher level or exclusively in female zebrafish (Supplementary Fig. S1). Interestingly, we found that some ribosomal proteins and other proteins responsible for protein synthesis were only detected in the livers of female zebrafish (Supplementary Table 1). This enhanced protein synthesis machinery aligns with the elevated demand for protein production during oogenesis, particularly for vitellogenins, which are produced in higher quantities in females in the present study (Supplementary Table 1), and essential for embryo nourishment^32,33^. A transcriptomics study showed higher levels of transcripts associated with several protein synthesis machinery components such as ribosomes, as well as vitellogenin, in the livers of female fish^13^.

On the other hand, females also exhibit a faster rate of liver regeneration^7^ that suggests a potential connection between enhanced protein synthesis capacity and regenerative ability in females. Given that liver regeneration requires massive protein synthesis for cellular proliferation and tissue remodeling, it is plausible that the transcriptional and translational resources primarily allocated for oogenesis can be repurposed and shifted for tissue repair following injury and give an advantage for liver regeneration in females.

Estradiol, an estrogen hormone synthesized in the ovaries of females, has been shown to induce production of vitellogenins in the liver, which serve as yolk proteins for oogenesis^34,35^. Additionally, liver regeneration is accelerated by estradiol^36^. The presence of a shared hormonal pathway for both oogenesis and liver regeneration further supports our hypothesis that the stronger protein synthesis machinery used during oogenesis may also contribute to the superior female liver regenerative capacity. Future experiments involving ovary ablation and analysis of its potential effects on the molecular patterns of liver protein synthesis, particularly those related to oogenesis and liver regeneration, could provide insights into the molecular mechanisms of sex differences in liver function, and help develop sex-tailored regenerative therapeutic approaches in humans.

### Metabolism and energy production

Our results show that proteins in metabolic pathways of energy production such as glycolysis, gluconeogenesis, the tricarboxylic acid (TCA) cycle, and β-oxidation are found at higher levels in the liver of male zebrafish (Fig. 2b, d, e). The upregulation of valine, leucine and isoleucine degradation pathway was observed in the liver of male zebrafish (Supplementary Fig. S2). In addition, upregulation of glycine, serine, and threonine metabolism, and pyruvate metabolism pathways were observed in the liver of male zebrafish (Supplementary Figs. S3, S4). In contrast to the female liver, which is programmed toward anabolic processes such as supporting high rates of protein and lipid synthesis for vitellogenesis and egg production (Fig. 2a, c, e), the male liver appears to channel more of its metabolic capacity toward energy production pathways. Earlier studies on the metabolome and transcriptome profiles of the fish liver showed upregulation of energy production pathways involved in the metabolism of carbohydrates, amino acids, and lipids in the livers of males compared to females^13,14,37^.

It has been shown that the biomass of gametes produced by females, ranging from invertebrates to mammals, are two to four times higher compared to males, indicating higher demands for materials needed for oogenesis in females^38^. Therefore, it could be hypothesized that because male reproductive physiology imposes relatively lower demands for spermatogenesis as compared to the huge investment required for oocyte production in females, their livers may naturally be able to spend more on pathways that generate ATP through the catabolism and oxidation of amino acids, carbohydrates, and lipids. For example, the liver of male zebrafish showed higher quantities of enzymes responsible for breaking down valine, leucine, and isoleucine, which are three essential and branched-chain amino acids necessary for protein synthesis. In other words, females down-regulate the catabolic pathways to save and redirect amino acids, proteins, and lipids toward the production of eggs and embryos. On the other hand, testosterone in males can upregulate energy production pathways such as the TCA, glycolysis, and β-oxidation^39^. Male fish engage in fighting with rival males, active courtship, and territorial defence^40,41^ which are energetically demanding physical activities and may partially explain our observation of the upregulation of energy production pathways in their livers. Future studies that perturb energy production pathways and observe their potential impacts on male-specific functions may shed light on the molecular basis of male sex-specific behaviour.

### Drug metabolism, detoxification, and antioxidant activity

When looking at individual proteins, we found significant sex-biased differences in the quantities of proteins and their isoforms involved in drug metabolism, detoxification, and antioxidant activity in the liver of zebrafish.

**Fig. 3:**
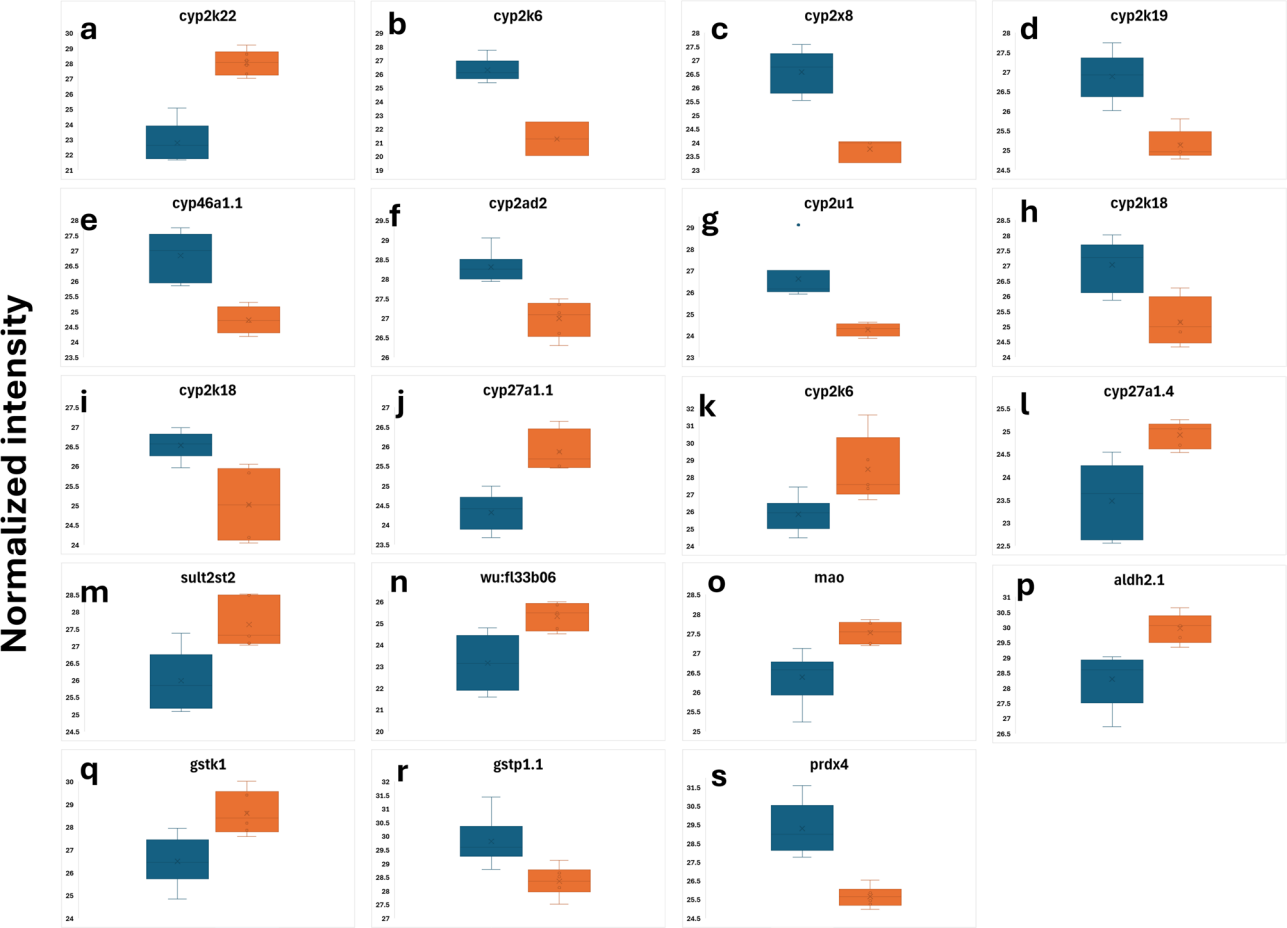
Normalized abundances (log2) of proteins related to drug metabolism, detoxification, and antioxidant activity, showing significant sex-biased differences in the zebrafish liver. Left and right boxes in each chart represent females and males, respectively.

For example, significant sex-biased differences were found in the quantities of several cytochrome p450 components in the liver (Fig. 3a–l). Three isoforms of cytochrome p450 were only detected in females. An isoform of UDP-glucuronosyltransferase was only detected in the liver of females (Supplementary Table 1). While one protein group including sult2st2 of sulfotransferase was found at higher levels in males (Fig. 3m), another protein group with sult3st3 and sult3st2 peptides was only detected in females (Supplementary Table 1). Flavin-containing monooxygenase and amine oxidase were higher in males (Fig. 3n, o). The level of aldehyde dehydrogenase, mitochondrial was higher in the liver of male zebrafish (Fig. 3p). While the level of glutathione S-transferase kappa 1 isoform X2 (gstk1) was higher in males (Fig. 3q), another isoform of glutathione S-transferase (gstp1.1) was found in a higher quantity in females (Fig. 3r). In addition, thioredoxin-dependent peroxiredoxin is detected in a higher quantity in the liver of females (Fig. 3s).

Sex-biased expression of genes related to drug metabolism, detoxification of xenobiotics, and antioxidant activity has already been shown at the transcriptome level in the liver^13^. Different levels of such proteins can affect the capacity of the liver in each sex to deal with drugs, toxicants, and oxidative stress. Earlier studies showed sex-biased trends in such proteins^42,43^. However, the application of deep mass spectrometry-based proteomics enabled us to generate a more detailed and higher-resolution list of relevant proteins compared to similar earlier studies.

In addition, isoforms of a protein may show different catalytic efficiencies and substrate specificities that may lead to unequal capacities for drug metabolism and detoxification between the sexes. These differences can significantly affect the pharmacokinetics of drugs, which can change their clearance rates, bioavailability, effectivity, and toxicity. Sex-based variations in enzyme isoforms are particularly important for drug design and toxicological assessments^44^. Future research should focus on characterizing sex as a biological variable in toxicological and pharmacological studies to improve personalized medicine approaches.

### Sex-biased levels of proteins in the liver and relationship with sex-biased hepatic diseases

We also observed sexual dimorphisms in the levels of multiple proteins known to be involved in hepatic diseases, which are summarized in Table 1.

**Table 1:** Sex-biased proteins in the liver of zebrafish and their potential roles in liver health and diseases.

Protein name	Trend	Role in disease	References
5’-3’ Exoribonuclease 1	OF	Suppression of HCV	Sedano et al.^45^; Li et al.^46^
Aldose reductase	M	Development of NAFLD	Sanchez-Lozada et al.^48^; Qiu and Guo^49^,
Alpha-2-HS-glycoprotein (fetuin)	M	Development of cancers and fatty liver diseases	Stefan et al.^51^; Ochieng et al.^52^
Aminoacyl tRNA synthetase complex-interacting multifunctional protein 1	F	Tumour-suppressive function	Zhou et al.^53^
C1q and TNF related 9 isoform X2	OF	Protection against NAFLD	Jung et al.^54^
Collagens	M, OM	Excessive collagen deposition in the liver is linked to NAFLD, fibrosis, cirrhosis and cancer.	Buzzetti et al.^55^; Karsdal et al.^56^; Ding et al.^57^
COP9 signalosome complex subunit 2	F	Action against fibrosis, cirrhosis and cancer via DNA repair.	Meir et al.^58^; Lei et al.^59^
Cytoglobin-1	F	Action against liver cancer	Thuy et al.^60,61^
DDRGK domain-containing protein 1	F	Action against liver fibrosis and carcinogenesis	Chen et al.^62^
E3 UFM1-protein ligase 1	F	Action against liver fibrosis and carcinogenesis	Chen et al.^62^
Death-associated protein 1 homolog	F	Action against progression of tumours	Wazir et al.^63^; Jia et al.^64^
DNA-(apurinic or apyrimidinic site) lyase	F	DNA damage repair and prevention of aging and cancer	Willis et al.^65^; Wallace^66^,
Filamin-B	M	Promotes HBV	Li et al.^67^
H/ACA ribonucleoprotein complex subunit DKC1	F	Action against hepatitis, NAFLD, and cancer by maintaining telomere integrity.	Garus and Autexier^70^, ; Donati^71^, ; Kim et al.^72^; Barnard et al.^73^
Integrin beta	M	Supporting progression of cancers, resistance against chemotherapy, and HCV and fibrosis.	Sun et al.^74^; Nejjari et al.^75^; Rahman et al.^76^
Myosin	M	Promotes NAFLD, HCV, fibrosis, and liver cancer.	Von Muhlen et al.^77^; Aref et al.^78^
Perilipin	OM	Promotes NAFLD	Orlicky et al.^79^
Phosphoethanolamine N-methyltransferase	F	Protection against NAFLD	Piras et al.^80^
Plastin-2	M	Overexpression in cancers	Shinomiya^81^,
Scinderin-like A	M	Promotes liver and other cancers.	Zhai et al.^82^; Wang and Luo^83^, ; Chen et al.^84^
Programmed cell death 4a, 5 and 6	F, OF	Tumour suppressors	Fu et al.^85^; Han et al.^86^
Selenoproteins S and F	F, OF	Protection against hepatic steatosis	Qiao et al.^87^; Zheng et al.^88^

M: male, F: female, OM: only in male, OF: only in female.

The 5’–3’ Exoribonuclease 1 was only detected in the liver of female zebrafish. It has been shown that this enzyme can suppress hepatitis C virus (HCV) via decaying its RNA^45,46^. A large systematic review of HCV epidemiology revealed a higher infection rate in males^47^. The exclusive presence of such enzymes in females can partially explain bias in the prevalence of HCV.

Aldose reductase was found at a higher level in the liver of male zebrafish. A relationship was detected between the level of this enzyme and the development of hepatic diseases such as NAFLD^48^. The inhibitor of this enzyme is proposed as a potential therapeutic approach against NAFLD^49^. The rates of NAFLD and HCC are higher in males^5,6^.

Alpha-2-HS-glycoprotein (fetuin) was found in higher quantities in the liver of male zebrafish. The testosterone is shown to upregulate the alpha-2-HS-glycoprotein transcription in the liver of males^50^. In addition, the level of this protein is positively associated with the accumulation of hepatic lipids and fatty liver diseases^51^ and the progression of different kinds of cancers^52^.

Aminoacyl tRNA synthetase complex-interacting multifunctional protein 1 showed higher quantities in the liver of female zebrafish. The tumour-suppressive function of this group of proteins has been documented in different types of cancers^53^.

C1q and TNF related 9 isoform X2 was only detected in the liver of female zebrafish. This protein functions against obesity and protects against NAFLD^54^.

Several types of collagens were more abundant in the liver of male zebrafish (Supplementary Table 1). Collagens play important roles in structure and function, as they are the primary component of the extracellular matrix. However, excessive collagen deposition in the liver is often linked to liver diseases, such as NAFLD, fibrosis, cirrhosis, and cancer^55–57^. It could be hypothesized that higher levels of collagen in the liver of males may predispose them to hepatic diseases and partially be responsible for higher rates of such diseases in males.

A higher level of COP9 signalosome complex subunit 2 was detected in the liver of female zebrafish. This group of proteins can repair DNA double-strand breaks^58^. DNA damages are the roots of many diseases, including cancer. In addition, deficiency of such proteins can lead to excessive apoptosis in the liver with subsequent risk of liver fibrosis and cirrhosis^59^.

The concentration of cytoglobin-1 was higher in the liver of female zebrafish. Cytoglobin is an oxygen carrier that acts against oxidative stress. Deficiency of cytoglobin can activate the oxidative stress pathway and promote the development of liver cancer^60,61^.

DDRGK domain-containing protein 1 (also called Ufbp1) and E3 UFM1-protein ligase 1 (also known as Ufl1), two endoplasmic reticulum-related proteins, were more abundant in the liver of female zebrafish. Loss of Ufl1/Ufbp1 in hepatocytes can promote pathological damages such as fibrosis and carcinogenesis^62^. This could imply that the protein quality control in the endoplasmic reticulum is stronger in female’s liver, enabling elimination of damaged or misfolded proteins that may cause some hepatic diseases, in line with the lower incidence of hepatic diseases in females. The functional enrichment analysis also showed enrichment of pathway for protein processing in the endoplasmic reticulum in the liver of female zebrafish (Fig. 2a).

A higher level of death-associated protein 1 homolog was quantified in the liver of female zebrafish. Studies showed an inverse correlation between the mRNA expression of this gene in tissue and progression of tumour in different cancer types^63,64^, indicating a potential protective function of this protein against the development of cancer.

DNA-(apurinic or apyrimidinic site) lyase was higher in the liver of female zebrafish. It is able to repair DNA damages caused by oxidative stress and prevent mutation^65^. Accumulation of DNA damages in cells can induce aging and cancer^66^.

Filamin-B was measured in a higher quantity in the liver of male zebrafish. This cytoskeleton protein can interact with the hepatitis B virus (HBV) and promote virus replication^67^. The epidemiological observations showed that HBV-induced hepatocarcinogenesis occurs more often in men than in women, with a 5–7:1 ratio^68^.

The quantity of H/ACA ribonucleoprotein complex subunit DKC1, also known as dyskerin, was higher in the liver of female zebrafish. Maintaining telomere integrity is one of the crucial roles of this protein. Lack of this enzyme can lead to the shortening of telomere^69,70^ and subsequently higher prevalences of hepatic diseases such as hepatitis, NAFLD, and cancer^71–73^.

Integrin beta showed a higher quantity in the liver of male zebrafish. This transmembrane protein mediates cell-cell and cell-extracellular matrix interactions. Integrin beta plays crucial roles in the progression of cancers and resistance against chemotherapy^74^. The abundance of this protein positively correlated with the progression of some chronic hepatic diseases, such as hepatitis C and fibrosis^75^. Integrins are being studied as a target for drug design in liver fibrosis^76^.

Myosin levels were higher in the liver of male zebrafish. Upregulation of myosin in hepatic diseases, such as NAFLD, HCV, fibrosis, and liver cancer candidates this protein as a target for therapy^77,78^.

Two isoforms of perilipin were only detected in the liver of male zebrafish. It was demonstrated that extra perilipin actions in the liver can promote NAFLD pathophysiology via effects on obesity, inflammation, and insulin resistance^79^.

Phosphoethanolamine N-methyltransferase is higher in the liver of female zebrafish. A reduced function of this protein has been linked to the increased risk of liver disease, including NAFLD^80^.

The concentration of plastin-2, also known as L-plastin, is higher in the liver of male zebrafish. This protein is an actin-binding protein that regulates the cytoskeleton. Overexpression of this protein has been recorded in several cancers^81^.

Scinderin-like A, a cytoskeleton protein, is found to be higher in the liver of male zebrafish. It was reported that scinderin was dramatically upregulated in liver cancer, and in vitro and in vivo knockdown of the gene can suppress cell proliferation in tumour^82^. Also, scinderin showed a cancer-promoting role in glioma^83^. In addition, suppression of scinderin impaired the progression of gastric cancer^84^.

The quantities of programmed cell death 4a and 5 are higher in the liver of female zebrafish, and programmed cell death 6 is only detected in the liver of female zebrafish. This protein group has been identified as potential tumour suppressor in various cancers, including HCC, via activation of apoptosis^85,86^.

Selenoprotein S is higher in the liver of female zebrafish, and selenoprotein F is only detectable in the liver of female zebrafish. Deficiency of hepatic selenoprotein S aggravates hepatic steatosis^87^. In addition, the knockout of selenoprotein F leads to glucose and lipid metabolism disorders, ultimately resulting in hepatic steatosis^88^. This indicates the involvement of selenoproteins as endoplasmic reticulum-associated proteins in maintaining metabolic homeostasis in the liver and protection against hepatic diseases.

Our data revealed that quantities of key proteins involved in both gluconeogenesis and glycolysis are significantly higher in the male liver compared to the female (Fig. 2b, d, e). A study in the liver of healthy rats showed higher levels of glycogen, higher expression of gluconeogenic genes, and glucose output in males^89^. The Warburg effect refers to the preference of cancer cells for glycolysis, which allows them to rapidly generate ATP and produce intermediates necessary for tumour progression^90^. While higher activities of gluconeogenesis and glycolysis pathways in male livers do not directly cause liver cancer, it could predispose males to liver cancer due to increased precursor availability and pro-tumour metabolic environment that may facilitate cancer initiation and progression. If similar protein level differences are found in humans, it could potentially give a clue for the higher prevalence of liver cancer in males than females.

Further mechanistic studies are necessary to reveal how increased male-biased gluconeogenesis and glycolysis interact with other oncogenic processes, and whether targeted modulation of these pathways could help mitigate HCC risk in vulnerable male populations.

## Conclusion

The present study reveals a wide sexual dimorphism in the liver proteome of zebrafish that highlights fundamental differences in protein, lipid, and carbohydrate metabolism between sexes. The female liver exhibits a greater capacity for protein synthesis, especially for oogenesis. In contrast, the male liver is more active in energy-producing pathways such as the TCA cycle, β-oxidation, and glycolysis. This suggests that females conserve their resources for reproductive functions such as gamete production, which is costlier for females than males, while males tend to turn their resources into energy. A higher protein synthesis capacity in females may potentially be used during liver injuries and enhance their regenerative abilities compared to males. Moreover, we identified significant differences in the abundance of proteins involved in drug metabolism that emphasize the necessity of considering sex as an important factor in toxicological and pharmacological studies and personalized medicine. The sex-biased differences in abundances of some proteins may predispose males to liver diseases while providing a protective advantage to females. The molecular insights from the zebrafish model can serve as a platform for future sex-specific mechanistic research that could provide tailored therapeutic strategies for human liver diseases and regenerative medicine.

## Supplementary Information

Below is the link to the electronic supplementary material.

Supplementary Material 1

Supplementary Material 2

## Data Availability

The mass spectrometry proteomics data have been deposited to the ProteomeXchange Consortium via the PRIDE partner repository with the dataset identifier PXD061886 and are available at https://www.ebi.ac.uk/pride/archive/projects/PXD061886.
