# Supplementary material for: Sexual dimorphism in zebrafish liver proteins and implications for hepatic regeneration and diseases
Source: Sci Rep. 2025 Sep 29;15:33565. doi: 10.1038/s41598-025-18599-2 (PMC12480104; doi:10.1038/s41598-025-18599-2)
Supplement: Supplementary file 1 — Supplementary Material 1 [file 41598_2025_18599_MOESM1_ESM.pdf]

Supporting information:

Sexual dimorphism in zebrafish liver proteins: Implications for hepatic regeneration and diseases

**Table of contents**	**Page**
Supplementary Methods	2
Supplementary Figure S1	3
Supplementary Figure S2	4
Supplementary Figure S3	5
Supplementary Figure S4	6

Supplementary Methods

Protein extraction and protein digestion

The extraction of proteins from the samples and protein digestion were performed based on the methods described earlier. The process started by mixing livers with 1% SDS and homogenizing them using an ice bath-equipped probe sonicator. The supernatants were obtained by centrifugation, and protein concentrations of samples were measured using the Micro-Lowry method.

Proteins were initially subjected to reduction using 10 mM dithiothreitol (DTT) for 45 minutes at room temperature. Following this step, alkylation was carried out with 40 mM iodoacetamide (IAA) for 45 minutes in the absence of light. To terminate the reaction, DTT was added to achieve a final concentration of 20 mM. For the subsequent purification and digestion steps, 50 µg of protein was processed using hydrophilic interaction liquid chromatography (HILIC) (ReSyn Biosciences, South Africa) for clean-up, coupled with automated on-bead protein digestion facilitated by the KingFisher Flex system (Thermo Fisher Scientific, Germany) in a 96-well format.

The automated process involved several key stages. Magnetic microspheres (at a 1:10 protein-to-bead ratio) were pre-incubated and equilibrated in an equilibration buffer containing 15% acetonitrile (ACN) and 100 mM ammonium acetate (NH4Ac) at pH 4.5. The protein samples were then introduced into a binding buffer (30% ACN, 200 mM NH4Ac, pH 4.5) to facilitate protein attachment to the HILIC beads. Non-specifically bound proteins were removed through two sequential washes with 95% ACN. Proteins bound to the beads were subsequently digested with trypsin (sequencing grade, Promega AB) at a protein-to-trypsin ratio of 20:1. The digestion was performed in 50 mM ammonium bicarbonate for 1 hour at 47°C. The resulting peptides were then recovered from the plate and dried using a SpeedVac system (Thermo Fisher Scientific, Germany) before undergoing C18 desalting.

The desalting procedure was conducted using BioPureSPN™ Mini, PROTO 300 C18 columns (The Nest Group, Inc., MA, USA). The columns were first equilibrated with 100 µl of a solution containing 70% ACN and 5% formic acid (FA). This was followed by conditioning with 100 µl of 5% FA. Peptides were resuspended in 100 µl of 5% FA and loaded onto the column. After washing the column with 100 µl of 5% FA, the purified peptides were eluted with 100 µl of a solution containing 50% ACN and 5% FA. All centrifugation steps during this procedure were carried out at 50×g for 2 minutes using an Eppendorf bench-top centrifuge. Finally, the purified peptides were dried and stored at -20°C for future use.

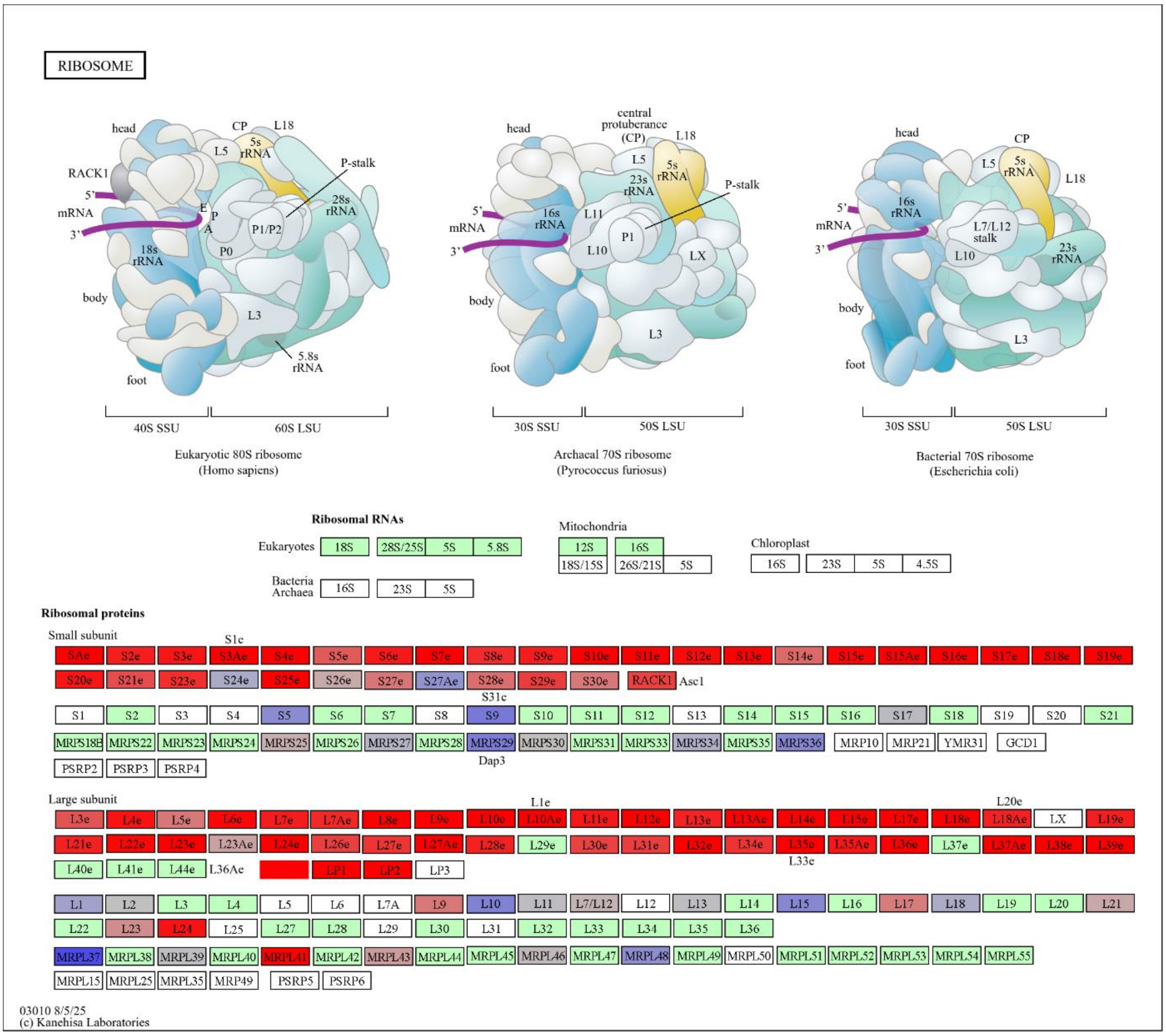

Supplementary Figure S1. KEGG [27-29] Ribosome (dre03010) with mapped proteins colored based on log2 fold change differences (saturation at log2 fold change 2), with red indicating higher abundance in female liver, blue higher in male liver, and grey no change. Red also includes proteins that were not detected in male zebrafish. Light green entries were not found in the present study and white entries had no mapped Zebrafish entry at KEGG. KEGG Mapper [30,31] was used for coloring.

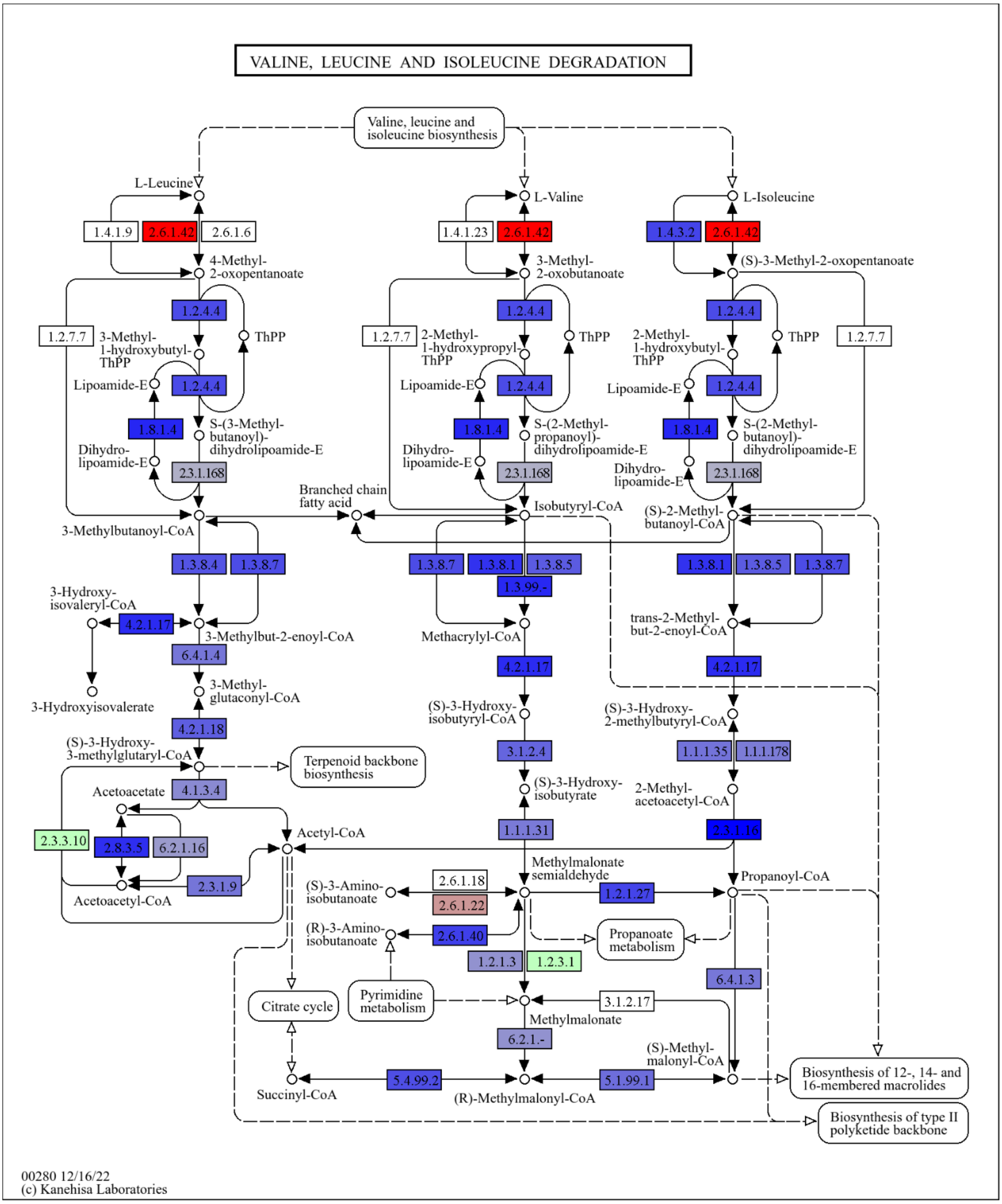

Supplementary Figure S2. KEGG [27-29] Valine, leucine, and isoleucine degradation pathway (dre00280) with mapped proteins colored based on log2 fold change differences (saturation at log2 fold change 2), with red indicating higher abundance in female liver, blue higher in male liver, and grey no change. Light green entries were not found in the present study and white entries had no mapped Zebrafish entry at KEGG. KEGG Mapper [30,31] was used for coloring.

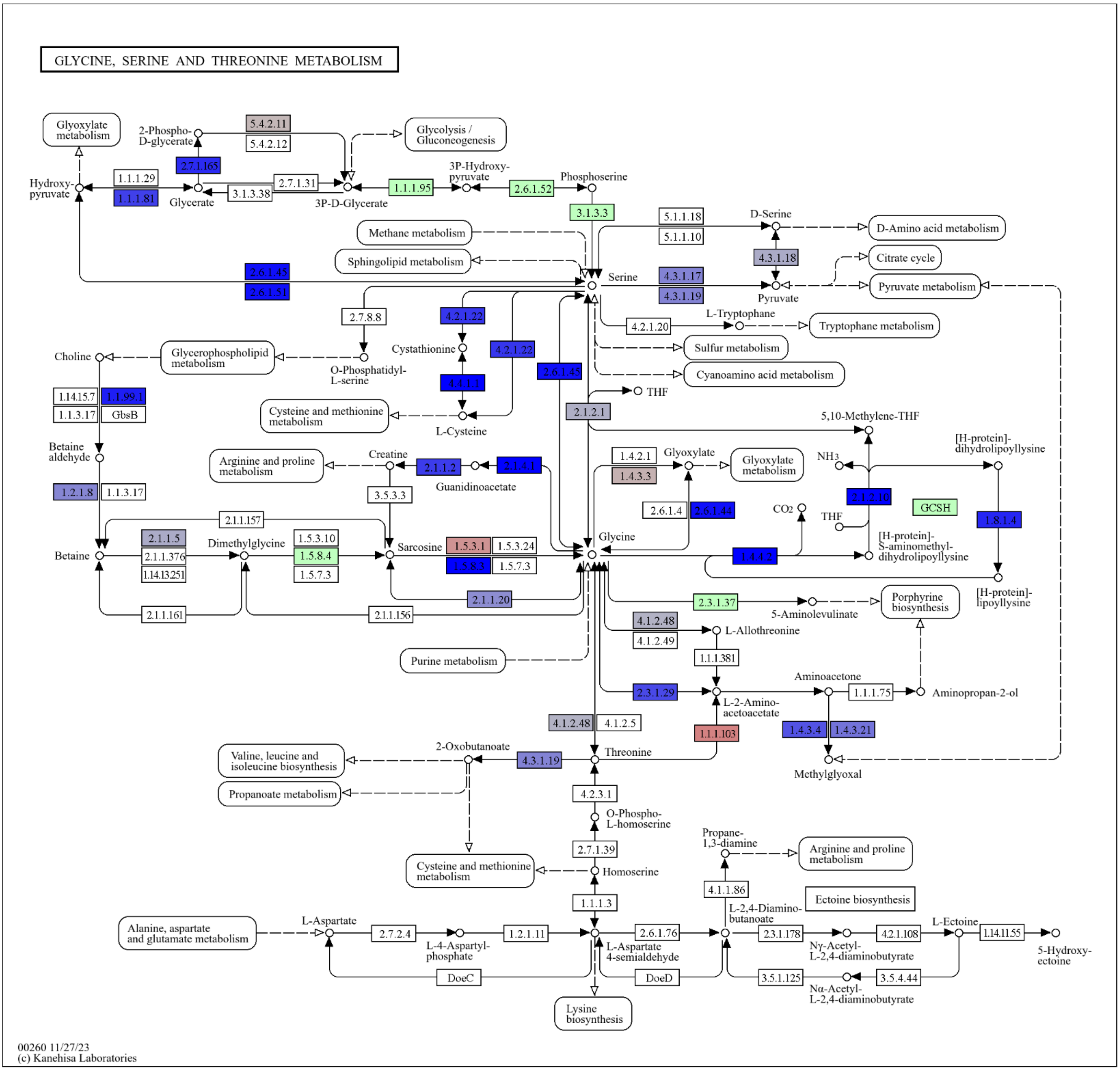

Supplementary Figure S3. KEGG [27-29] glycine, serine and threonine metabolism (dre00260) with mapped proteins colored based on log2 fold change differences (saturation at log2 fold change 2), with red indicating higher abundance in female liver, blue higher in male liver, and grey no change. Light green entries were not found in the present study and white entries had no mapped Zebrafish entry at KEGG. KEGG Mapper [30,31] was used for coloring.

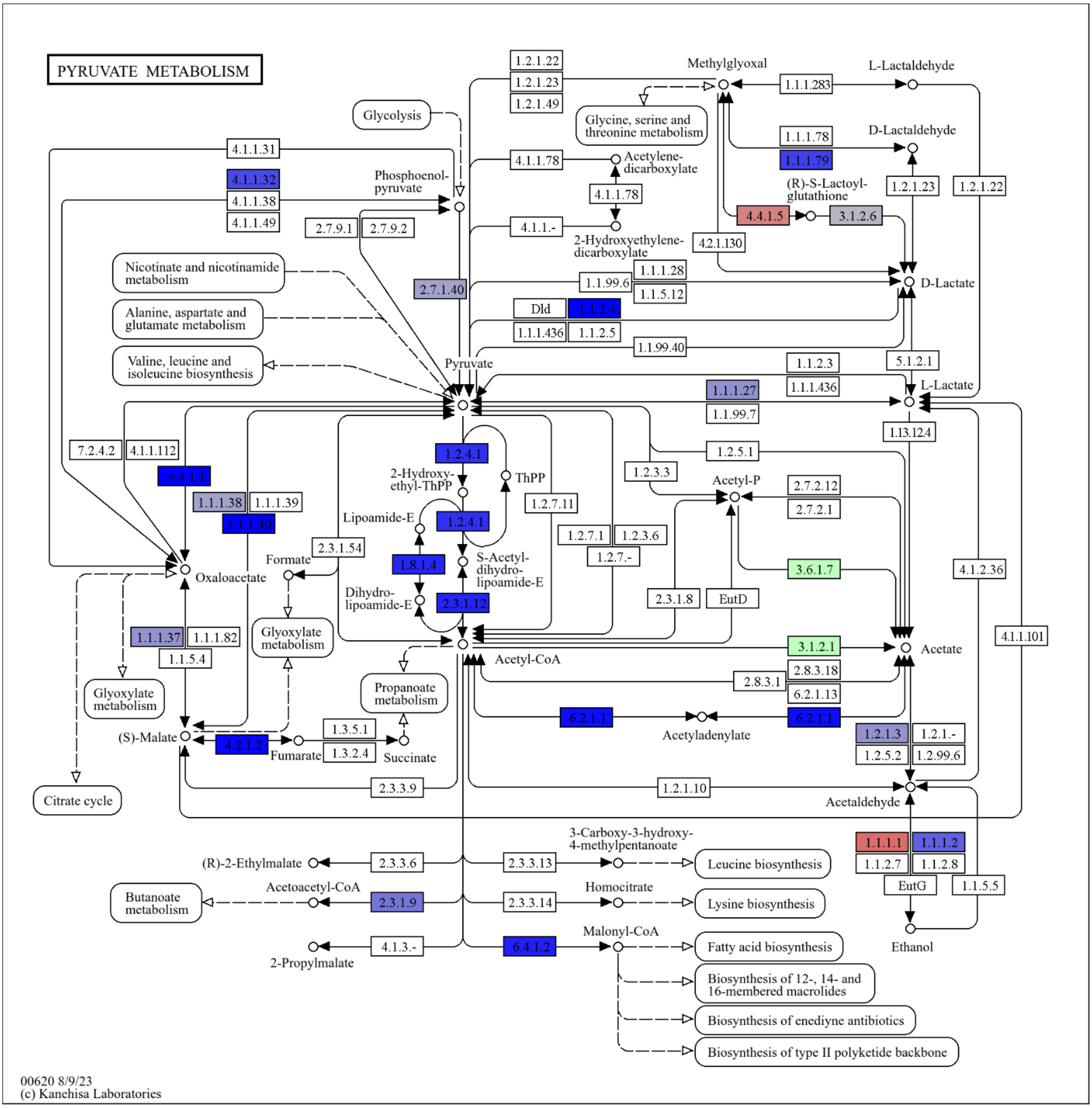

Supplementary Figure S4. KEGG [27-29] pyruvate metabolism (dre00620) with with mapped proteins colored based on log2 fold change differences (saturation at log2 fold change 2), with red indicating higher abundance in female liver, blue higher in male liver, and grey no change. Light green entries were not found in the present study and white entries had no mapped Zebrafish entry at KEGG. KEGG Mapper [30,31] was used for coloring.
